# Polymyxin-induced neuromuscular weakness: a case report

**DOI:** 10.3389/fneur.2024.1342419

**Published:** 2024-03-27

**Authors:** Amanda X. Y. Chin, Kay W. P. Ng, Yee Cheun Chan, Yihui Goh, Rahul Rathakrishnan

**Affiliations:** ^1^Division of Neurology, Department of Medicine, National University Hospital, Singapore, Singapore; ^2^Department of Medicine, Yong Loo Lin School of Medicine, National University of Singapore, Singapore, Singapore

**Keywords:** polymyxin, neuromuscular blockade, neurotoxicity, respiratory failure, multi-drug resistance, renal impairment

## Abstract

Polymyxin-induced neuromuscular blockade is a rare but potentially fatal condition, with majority of cases that were reported between 1962 and 1973. We describe a patient who developed hypercapnic respiratory failure after initiation of polymyxin for multi-drug resistant *Escherichia Coli* bacteremia, due to polymyxin-induced neuromuscular dysfunction. After cessation of polymyxin, he regained full strength, had complete resolution of ptosis, and was successfully extubated. In light of the renewed use of polymyxin in this era of antimicrobial-resistance, this case aims to raise awareness about this rare but life-threatening condition, which is easily reversible with early recognition and prompt discontinuation of the drug.

## 1 Introduction

Polymyxins were first discovered in 1947 by Japanese scientist Koyama, from *Paenibacillus polymyxa* (1). Polymyxin B and Polymyxin E (otherwise known as Colistin) were introduced for clinical use in the 1950s, but fell out of favour from the 1970s to 2000s in view of their significant risks of nephrotoxicity and neurotoxicity (1). However, in recent years, with the emergence of multi-drug resistant gram-negative bacilli, the use of polymyxins have renewed relevance, especially in the treatment of nosocomial infections (2). Polymyxin-neurotoxicity can manifest with paraesthesia, polyneuropathy, ataxia, giddiness, visual disturbances, neuromuscular blockade, confusion and convulsions (3, 4). Majority of cases with polymyxin-induced neuromuscular dysfunction were reported between 1962 and 1973. Here we describe a patient who developed drowsiness, ptosis and proximal weakness with fatigability secondary to polymyxin-induced neuromuscular blockade.

## 2 Case report

A 62-year-old male with diabetes mellitus, non-ischemic cardiomyopathy and chronic kidney disease was admitted for *Escherichia coli* bacteraemia secondary to cholecystitis. He received empirical intravenous ceftriaxone and metronidazole, which was switched to intravenous polymyxin B and tigecycline when blood cultures grew multi-drug resistant *Escherichia Coli*. He received polymyxin B at a loading dose of 25,000 IU/kg and a maintenance dose of 1,000,000 units 12 hourly (~14,705.9 IU/kg).

Three days later, he was intermittently drowsy. Computed tomography of the brain was unremarkable. The following day, he developed hypercapnic respiratory failure and hypoxic arrest requiring intubation and admission to the intensive care unit (ICU). He was extubated two days after, but developed recurrent hypercapnia and hypoxic arrest a few minutes after extubation. Chest radiograph was normal. As there was no apparent cardio-respiratory cause for his recurrent hypercapnia and drowsiness, a neurological consult was obtained.

**Figure 1:**
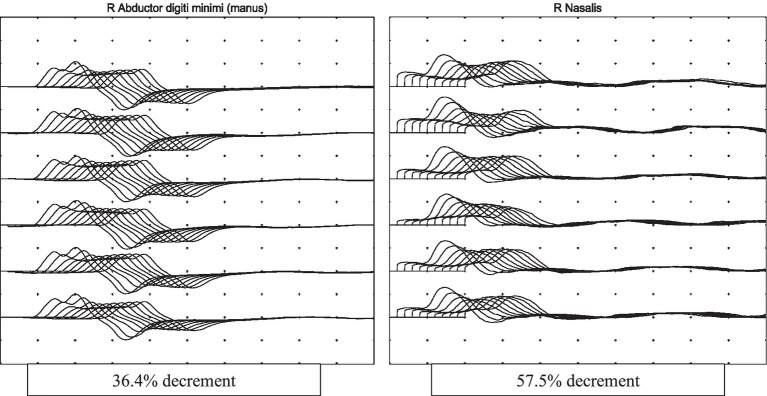
Repetitive nerve stimulation study done at 3 Hz shows abnormal decremental response in the muscles tested with characteristic U-shape envelope pattern, suggestive of a neuromuscular junction transmission defect.

On review by the Neurologist, the patient was on a low dose of Fentanyl infusion (30 mcg/h) for tube tolerance and was cooperative with testing. Neurological examination revealed generalised hyporeflexia, bilateral ptosis, neck flexion weakness and fatigable proximal limb weakness (proximal power MRC grade 1, distal power MRC grade 4) with no sensory loss. The rest of the neurological examination was unremarkable. The clinical findings were suggestive of a neuromuscular junction pathology. Repetitive nerve stimulation study during low-frequency stimulation demonstrated abnormal decremental response of more than 10% in the right abductor digitorum minimi, bilateral nasalis and left trapezius, and characteristic U-shaped envelope pattern, whereas high-frequency stimulation did not lead to any abnormal facilitation response in the muscles tested. This was consistent with a defect in post-synaptic neuromuscular junction transmission (see Figure 1). Creatine kinase (33 U/L) and aldolase (3.3 U/L) were normal. A review of his renal function at that point also showed an increase of blood creatinine levels from a baseline of 170 umol/L to 259 umol/L (eGFR 36 mL/min to 22 mL/min).

**Figure 2:**
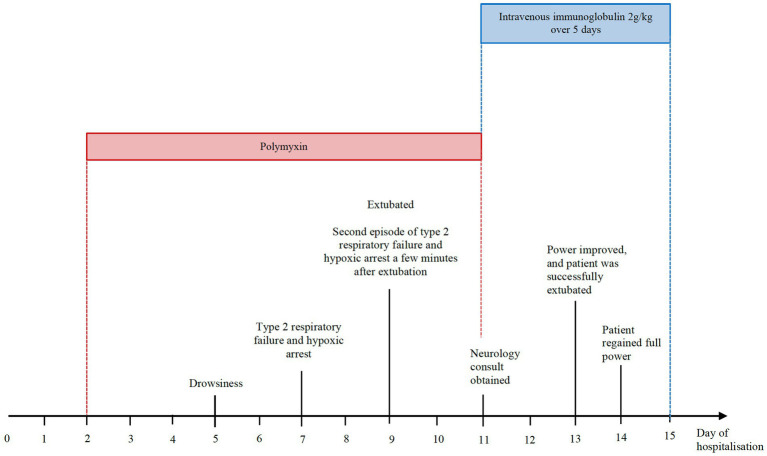
Timeline of events during hospitalisation.

Polymyxin was discontinued, and the patient was given intravenous immunoglobulin (IVIg) 2 g/kg/day over 5 days in view of the differential diagnosis of a myasthenic crisis unmasked by polymyxin. Within the next 48 h, his strength rapidly improved and he was extubated successfully. He regained full strength and ptosis resolved fully the following day (see Figure 2).

Interval repetitive nerve stimulation study that was repeated at this point normalised and serum acetylcholine receptor antibodies that were sent earlier returned negative. Additional history taken from the patient after recovery also revealed that he had no prior episodes of weakness or ptosis before this.

## 3 Discussion

Polymyxins are bactericidal agents that act via binding to the lipopolysaccharides and phospholipids of the membrane walls of gram-negative bacteria. By doing so, calcium and magnesium cations are competitively displaced, resulting in changes in permeability and disruption of the cell membrane (2). The prevalence of nephrotoxicity from polymyxins range from 20 to 60%, whereas that of polymyxin-induced neurotoxicity is estimated to be approximately 7% based on a prospective study that included 317 courses of colistin (3–5). Various neurological adverse effects include paraesthesia, polyneuropathy, ataxia, giddiness, visual disturbances, neuromuscular blockade, confusion and convulsions (3, 4). Neurotoxicity is postulated to arise due to the interaction of polymyxins with lipid-rich neurons and exhibits a dose-dependent relationship (4).

Polymyxin can cause neuromuscular blockade at both pre-and post-synaptic sites, leading to fatigability which can occur as quickly as 1 to 26 h after receiving polymyxin (6). A form of pre-synaptic dysfunction occurs due to non-competitive blockade, which results in reduced acetylcholine being released into the synaptic gap (6). Another proposed mechanism is that of calcium depletion, which leads to a prolonged depolarisation phase (7). *In vitro* studies have also shown that polymyxin B can also result in post-synaptic dysfunction as it can reduce membrane sensitivity to acetylcholine (8–10).

The International Consensus Guidelines for the Optimal Use of the Polymyxins recommends a loading dose of 2.0–2.5 mg/kg (equivalent to 20,000–25,000 IU/kg) and maintenance dose of 1.25–1.5 mg/kg (equivalent to 12,500–15,000 IU/kg total body weight) every 12 h (11). The guidelines suggest that in a patient with renal impairment, dose reduction is not necessary. This is based on pharmacokinetic studies that show that the clearance of polymyxin B is not affected by creatinine clearance, as majority of the drug is not eliminated via the kidneys (11).

Despite an appropriate dose given, our patient developed hypercapnic respiratory failure secondary to post-synaptic neuromuscular blockade and weakness following polymyxin use. A significant contributor to polymyxin neurotoxicity was the patient’s underlying chronic kidney disease which worsened as a result of sepsis and polymyxin. Elevated risks of neuromuscular junction dysfunction and respiratory paralysis with polymyxin have been reported in patients with renal dysfunction (acute or chronic), and myasthenia gravis (4, 12).

Other risk factors for polymyxin-neurotoxicity include hypoxia, higher doses or prolonged durations of treatment, and female gender (4, 5, 13). Concomitant administration of drugs such as narcotics, sedatives, anaesthetic drugs, corticosteroids or muscle-relaxants have also been described to increase the risk of neurotoxic events (4, 14). Of note, our patient did not receive any of these medications and was only kept on low dose intravenous fentanyl of 30 mcg/h for tube tolerance in the intensive care unit.

Timely recognition of fatigable weakness as a cause of his recurrent decompensated respiratory failure resulted in cessation of polymyxin in our patient. This allowed him to have full neurological recovery. Given the possibility of underlying undiagnosed myasthenia gravis that was unmasked by polymyxin, he was also started on concurrent intravenous immunoglobulin (IVIg) therapy. However, this was unlikely to have contributed significantly to neurological improvement as he had shown signs of improvement 48 h after cessation of polymyxin B, and regained full power even before completion of his full course of IVIg. 4 years later, he has remained well without treatment following the cessation of polymyxin.

The mainstay of management of polymyxin neurotoxicity and neuromuscular weakness comprises early recognition and prompt discontinuation of the drug, coupled with supportive therapy such as mechanical ventilation for respiratory failure. Stopping the causative agent is crucial as the adverse effects are usually reversible once it is withdrawn (4, 15–17). Cholinesterase inhibitors such as neostigmine have not been proven to be effective, and this is presumed to be due to the effects of non-competitive blockade at the myoneural end plate resulting in pre-synaptic dysfunction (7). The administration of intravenous calcium has also been attempted but this has variable results (4, 6). Renal replacement therapy has been used to aid with colistin removal in cases of renal impairment, although this appears to be less efficacious for polymyxin B, where only 6–12% of the drug is removed by continuous renal replacement therapy (18).

## 4 Conclusion

While few studies have highlighted weakness and respiratory failure from neuromuscular blockade as adverse effects of polymyxin, majority of these cases were reported between 1962 and 1973 (6, 12, 14–17). With the renewed use of polymyxin in the era of antimicrobial-resistance, physicians should have heightened awareness of this potentially serious complication. To avoid the adverse effects of polymyxin, it is essential that an appropriate dose is given, coupled with close monitoring of the patient’s renal function and neurological status during the course of therapy. Recognition and prompt discontinuation of polymyxin is essential given the easily reversible nature of this life-threatening condition.

## Data availability statement

The original contributions presented in the study are included in the article/supplementary material, further inquiries can be directed to the corresponding author.

## Ethics statement

Written informed consent was obtained from the individual(s) for the publication of any potentially identifiable images or data included in this article.

## Author contributions

AC: Conceptualization, Formal analysis, Writing – original draft, Writing – review & editing. KN: Formal analysis, Writing – review & editing. YC: Formal analysis, Writing – review & editing. YG: Formal analysis, Writing – review & editing. RR: Formal analysis, Writing – review & editing.
